# Supplementary material for: Modeling glioblastoma heterogeneity as a dynamic network of cell states
Source: Mol Syst Biol. 2021 Sep 16;17(9):e10105. doi: 10.15252/msb.202010105 (PMC8444284; doi:10.15252/msb.202010105)
Supplement: Supplementary file 5 — Source Data for Figure 3 [file MSB-17-e10105-s001.zip › Figure3A_sourcedata/GSEA_3065/hallmarks_state3.GseaPreranked.1623416274744/HALLMARK_ADIPOGENESIS.html]

Details for gene set HALLMARK\_ADIPOGENESIS[GSEA]

|  || Dataset | state3 |
| Phenotype | NoPhenotypeAvailable |
| Upregulated in class | na\_neg |
| GeneSet | HALLMARK\_ADIPOGENESIS |
| Enrichment Score (ES) | -0.34379455 |
| Normalized Enrichment Score (NES) | -1.3040148 |
| Nominal p-value | 0.04888889 |
| FDR q-value | 0.1802313 |
| FWER p-Value | 0.922 |
Table: GSEA Results Summary

  

Fig 1: Enrichment plot: HALLMARK\_ADIPOGENESIS      
 Profile of the Running ES Score & Positions of GeneSet Members on the Rank Ordered List

  

| PROBE | GENE SYMBOL | GENE\_TITLE | RANK IN GENE LIST | RANK METRIC SCORE | RUNNING ES | CORE ENRICHMENT || 1 | APLP2 |  |  | 20 | 0.506 | 0.0246 | No |
| 2 | RTN3 |  |  | 50 | 0.435 | 0.0443 | No |
| 3 | LPL |  |  | 68 | 0.381 | 0.0625 | No |
| 4 | ATP1B3 |  |  | 114 | 0.330 | 0.0748 | No |
| 5 | UBC |  |  | 145 | 0.313 | 0.0880 | No |
| 6 | POR |  |  | 239 | 0.269 | 0.0914 | No |
| 7 | COL4A1 |  |  | 241 | 0.267 | 0.1055 | No |
| 8 | DHRS7 |  |  | 323 | 0.238 | 0.1087 | No |
| 9 | PPP1R15B |  |  | 399 | 0.219 | 0.1116 | No |
| 10 | REEP5 |  |  | 429 | 0.213 | 0.1196 | No |
| 11 | LAMA4 |  |  | 496 | 0.199 | 0.1225 | No |
| 12 | ITGA7 |  |  | 595 | 0.187 | 0.1210 | No |
| 13 | PHLDB1 |  |  | 606 | 0.185 | 0.1297 | No |
| 14 | ACLY |  |  | 624 | 0.183 | 0.1375 | No |
| 15 | CD151 |  |  | 629 | 0.182 | 0.1467 | No |
| 16 | DHCR7 |  |  | 669 | 0.176 | 0.1515 | No |
| 17 | IFNGR1 |  |  | 733 | 0.166 | 0.1530 | No |
| 18 | DHRS7B |  |  | 766 | 0.161 | 0.1579 | No |
| 19 | ADCY6 |  |  | 865 | 0.151 | 0.1545 | No |
| 20 | ABCB8 |  |  | 866 | 0.151 | 0.1626 | No |
| 21 | PTCD3 |  |  | 947 | 0.143 | 0.1608 | No |
| 22 | ELOVL6 |  |  | 1050 | 0.134 | 0.1561 | No |
| 23 | ESYT1 |  |  | 1124 | 0.128 | 0.1544 | No |
| 24 | LIFR |  |  | 1260 | 0.117 | 0.1450 | No |
| 25 | SLC5A6 |  |  | 1310 | 0.115 | 0.1453 | No |
| 26 | SLC19A1 |  |  | 1494 | 0.103 | 0.1296 | No |
| 27 | RMDN3 |  |  | 1514 | 0.102 | 0.1328 | No |
| 28 | ACOX1 |  |  | 1544 | 0.101 | 0.1348 | No |
| 29 | GPAT4 |  |  | 1546 | 0.101 | 0.1400 | No |
| 30 | SCARB1 |  |  | 1789 | 0.087 | 0.1165 | No |
| 31 | RIOK3 |  |  | 1799 | 0.087 | 0.1201 | No |
| 32 | ALDH2 |  |  | 1885 | 0.082 | 0.1145 | No |
| 33 | SLC27A1 |  |  | 1928 | 0.079 | 0.1139 | No |
| 34 | HIBCH |  |  | 1943 | 0.079 | 0.1164 | No |
| 35 | STOM |  |  | 2035 | 0.074 | 0.1098 | No |
| 36 | NKIRAS1 |  |  | 2076 | 0.073 | 0.1090 | No |
| 37 | ANGPT1 |  |  | 2102 | 0.072 | 0.1100 | No |
| 38 | DNAJB9 |  |  | 2115 | 0.071 | 0.1124 | No |
| 39 | ABCA1 |  |  | 2275 | 0.065 | 0.0973 | No |
| 40 | IDH1 |  |  | 2376 | 0.062 | 0.0890 | No |
| 41 | ATL2 |  |  | 2520 | 0.056 | 0.0754 | No |
| 42 | GPD2 |  |  | 2606 | 0.053 | 0.0683 | No |
| 43 | DLD |  |  | 2811 | 0.047 | 0.0471 | No |
| 44 | DGAT1 |  |  | 2890 | 0.044 | 0.0404 | No |
| 45 | ADIPOR2 |  |  | 3051 | 0.040 | 0.0239 | No |
| 46 | IMMT |  |  | 3080 | 0.039 | 0.0227 | No |
| 47 | AIFM1 |  |  | 3137 | 0.038 | 0.0182 | No |
| 48 | BCL6 |  |  | 3186 | 0.036 | 0.0145 | No |
| 49 | COQ3 |  |  | 3254 | 0.034 | 0.0086 | No |
| 50 | DBT |  |  | 3290 | 0.033 | 0.0063 | No |
| 51 | JAGN1 |  |  | 3325 | 0.032 | 0.0040 | No |
| 52 | G3BP2 |  |  | 3475 | 0.029 | -0.0118 | No |
| 53 | PPM1B |  |  | 3490 | 0.028 | -0.0119 | No |
| 54 | BCL2L13 |  |  | 3585 | 0.026 | -0.0214 | No |
| 55 | MIGA2 |  |  | 3720 | 0.022 | -0.0358 | No |
| 56 | MAP4K3 |  |  | 3856 | 0.019 | -0.0505 | No |
| 57 | CCNG2 |  |  | 4001 | 0.015 | -0.0665 | No |
| 58 | PDCD4 |  |  | 4013 | 0.015 | -0.0670 | No |
| 59 | CPT2 |  |  | 4059 | 0.014 | -0.0715 | No |
| 60 | PGM1 |  |  | 4098 | 0.013 | -0.0752 | No |
| 61 | MCCC1 |  |  | 4116 | 0.012 | -0.0766 | No |
| 62 | ELMOD3 |  |  | 4168 | 0.011 | -0.0819 | No |
| 63 | BAZ2A |  |  | 4310 | 0.006 | -0.0980 | No |
| 64 | SSPN |  |  | 4525 | 0.001 | -0.1228 | No |
| 65 | GBE1 |  |  | 4604 | -0.001 | -0.1318 | No |
| 66 | AGPAT3 |  |  | 4670 | -0.003 | -0.1392 | No |
| 67 | SAMM50 |  |  | 4711 | -0.004 | -0.1437 | No |
| 68 | UBQLN1 |  |  | 4740 | -0.004 | -0.1467 | No |
| 69 | LPCAT3 |  |  | 4822 | -0.007 | -0.1557 | No |
| 70 | DNAJC15 |  |  | 4921 | -0.009 | -0.1667 | No |
| 71 | NMT1 |  |  | 4988 | -0.011 | -0.1738 | No |
| 72 | DLAT |  |  | 5005 | -0.011 | -0.1750 | No |
| 73 | SLC25A10 |  |  | 5075 | -0.013 | -0.1824 | No |
| 74 | SPARCL1 |  |  | 5152 | -0.014 | -0.1905 | No |
| 75 | DRAM2 |  |  | 5296 | -0.018 | -0.2061 | No |
| 76 | PIM3 |  |  | 5307 | -0.018 | -0.2063 | No |
| 77 | MGST3 |  |  | 5395 | -0.021 | -0.2153 | No |
| 78 | ITSN1 |  |  | 5427 | -0.022 | -0.2177 | No |
| 79 | HADH |  |  | 5434 | -0.022 | -0.2173 | No |
| 80 | CAT |  |  | 5510 | -0.024 | -0.2247 | No |
| 81 | ESRRA |  |  | 5526 | -0.025 | -0.2251 | No |
| 82 | ARAF |  |  | 5713 | -0.030 | -0.2451 | No |
| 83 | PLIN2 |  |  | 5856 | -0.034 | -0.2598 | No |
| 84 | ACO2 |  |  | 5970 | -0.038 | -0.2709 | No |
| 85 | PEMT |  |  | 5982 | -0.039 | -0.2701 | No |
| 86 | CS |  |  | 5985 | -0.039 | -0.2683 | No |
| 87 | IDH3G |  |  | 6007 | -0.040 | -0.2686 | No |
| 88 | FAH |  |  | 6077 | -0.042 | -0.2744 | No |
| 89 | SORBS1 |  |  | 6085 | -0.042 | -0.2730 | No |
| 90 | GRPEL1 |  |  | 6088 | -0.042 | -0.2709 | No |
| 91 | UQCRC1 |  |  | 6149 | -0.045 | -0.2755 | No |
| 92 | ACADM |  |  | 6280 | -0.049 | -0.2880 | No |
| 93 | MYLK |  |  | 6330 | -0.050 | -0.2911 | No |
| 94 | PREB |  |  | 6379 | -0.052 | -0.2939 | No |
| 95 | ALDOA |  |  | 6448 | -0.054 | -0.2989 | No |
| 96 | TST |  |  | 6507 | -0.057 | -0.3026 | No |
| 97 | PFKL |  |  | 6771 | -0.070 | -0.3295 | No |
| 98 | SDHC |  |  | 6808 | -0.071 | -0.3299 | No |
| 99 | NDUFS3 |  |  | 6821 | -0.072 | -0.3275 | No |
| 100 | SLC25A1 |  |  | 6932 | -0.077 | -0.3361 | No |
| 101 | TALDO1 |  |  | 6943 | -0.078 | -0.3332 | No |
| 102 | DECR1 |  |  | 6981 | -0.079 | -0.3332 | No |
| 103 | TANK |  |  | 6995 | -0.080 | -0.3305 | No |
| 104 | SUCLG1 |  |  | 7007 | -0.082 | -0.3274 | No |
| 105 | PHYH |  |  | 7066 | -0.085 | -0.3297 | No |
| 106 | TOB1 |  |  | 7088 | -0.086 | -0.3275 | No |
| 107 | ETFB |  |  | 7090 | -0.086 | -0.3230 | No |
| 108 | COQ5 |  |  | 7098 | -0.087 | -0.3192 | No |
| 109 | UCK1 |  |  | 7181 | -0.092 | -0.3239 | No |
| 110 | IDH3A |  |  | 7286 | -0.099 | -0.3307 | No |
| 111 | COQ9 |  |  | 7291 | -0.100 | -0.3259 | No |
| 112 | YWHAG |  |  | 7373 | -0.106 | -0.3296 | No |
| 113 | QDPR |  |  | 7488 | -0.116 | -0.3367 | No |
| 114 | ACAA2 |  |  | 7550 | -0.122 | -0.3373 | Yes |
| 115 | SDHB |  |  | 7565 | -0.123 | -0.3324 | Yes |
| 116 | CDKN2C |  |  | 7609 | -0.128 | -0.3305 | Yes |
| 117 | PRDX3 |  |  | 7649 | -0.132 | -0.3280 | Yes |
| 118 | CRAT |  |  | 7655 | -0.133 | -0.3215 | Yes |
| 119 | ECH1 |  |  | 7658 | -0.134 | -0.3146 | Yes |
| 120 | MRPL15 |  |  | 7750 | -0.146 | -0.3175 | Yes |
| 121 | MGLL |  |  | 7773 | -0.148 | -0.3121 | Yes |
| 122 | CYC1 |  |  | 7816 | -0.154 | -0.3088 | Yes |
| 123 | ARL4A |  |  | 7829 | -0.155 | -0.3020 | Yes |
| 124 | CMBL |  |  | 7837 | -0.156 | -0.2945 | Yes |
| 125 | NDUFAB1 |  |  | 7858 | -0.160 | -0.2883 | Yes |
| 126 | UCP2 |  |  | 7873 | -0.162 | -0.2813 | Yes |
| 127 | CMPK1 |  |  | 7905 | -0.167 | -0.2761 | Yes |
| 128 | SCP2 |  |  | 7919 | -0.170 | -0.2685 | Yes |
| 129 | NDUFA5 |  |  | 7986 | -0.183 | -0.2665 | Yes |
| 130 | RNF11 |  |  | 7997 | -0.184 | -0.2578 | Yes |
| 131 | DDT |  |  | 8010 | -0.187 | -0.2493 | Yes |
| 132 | AK2 |  |  | 8036 | -0.191 | -0.2421 | Yes |
| 133 | GHITM |  |  | 8116 | -0.207 | -0.2402 | Yes |
| 134 | RAB34 |  |  | 8197 | -0.227 | -0.2374 | Yes |
| 135 | CAVIN1 |  |  | 8208 | -0.231 | -0.2263 | Yes |
| 136 | MTCH2 |  |  | 8221 | -0.234 | -0.2153 | Yes |
| 137 | CHCHD10 |  |  | 8233 | -0.235 | -0.2041 | Yes |
| 138 | MDH2 |  |  | 8251 | -0.242 | -0.1932 | Yes |
| 139 | NDUFB7 |  |  | 8276 | -0.248 | -0.1828 | Yes |
| 140 | VEGFB |  |  | 8283 | -0.254 | -0.1700 | Yes |
| 141 | GADD45A |  |  | 8306 | -0.260 | -0.1587 | Yes |
| 142 | ECHS1 |  |  | 8325 | -0.264 | -0.1468 | Yes |
| 143 | COX7B |  |  | 8447 | -0.314 | -0.1442 | Yes |
| 144 | SOD1 |  |  | 8526 | -0.349 | -0.1347 | Yes |
| 145 | ATP5PO |  |  | 8581 | -0.386 | -0.1205 | Yes |
| 146 | UQCR10 |  |  | 8608 | -0.410 | -0.1017 | Yes |
| 147 | COX8A |  |  | 8619 | -0.422 | -0.0804 | Yes |
| 148 | UQCR11 |  |  | 8686 | -0.503 | -0.0614 | Yes |
| 149 | UQCRQ |  |  | 8726 | -0.602 | -0.0339 | Yes |
| 150 | GPX4 |  |  | 8742 | -0.690 | 0.0010 | Yes |
Table: GSEA details [plain text format]

  

Fig 2: HALLMARK\_ADIPOGENESIS: Random ES distribution      
 Gene set null distribution of ES for **HALLMARK\_ADIPOGENESIS**
